# Toxic Epidermal Necrolysis Mimicking Severe Acute Graft-Versus-Host Disease After Allogeneic Hematopoietic Stem Cell Transplantation: A Diagnostic Challenge

**DOI:** 10.3390/jcm15124730

**Published:** 2026-06-18

**Authors:** Titas Tiškevičius, Egidija Kukarskytė, Ignas Gaidamavičius, Miglė Kulbokė, Martyna Beitnerienė, Rūta Dambrauskienė, Milda Rudžianskienė, Rima Jūratė Gerbutavičienė, Audronė Vaitiekienė, Rolandas Gerbutavičius, Domas Vaitiekus

**Affiliations:** 1 Department of Oncology and Hematology, Hospital of Lithuanian University of Health Sciences, Kaunas Clinics, 50161 Kaunas, Lithuania; 2 Medical Academy, Lithuanian University of Health Sciences, 44307 Kaunas, Lithuania; 3 Department of Immunology and Allergology, Hospital of Lithuanian University of Health Sciences, Kaunas Clinics, 50161 Kaunas, Lithuania; 4 Department of Drug Technology and Social Pharmacy, Lithuanian University of Health Sciences, 44307 Kaunas, Lithuania; 5 Department of Cardiology, Hospital of Lithuanian University of Health Sciences, Kaunas Clinics, 50161 Kaunas, Lithuania

**Keywords:** toxic epidermal necrolysis, graft-versus-host disease, hematopoietic stem cell transplantation, trimethoprim/sulfamethoxazole, severe cutaneous adverse reactions

## Abstract

**Background:** Toxic epidermal necrolysis (TEN) is a rare but life-threatening complication that may occur in patients after allogeneic hematopoietic stem cell transplantation (allo-HSCT), particularly in the context of extensive drug exposure. In this population, TEN can closely resemble severe acute graft-versus-host disease (GVHD), making diagnosis and management challenging. **Case presentation:** We report the clinical course of an allo-HSCT recipient who developed a rapidly progressive skin rash early after transplantation, and we analyzed the clinical features, histopathology, treatment and outcome. **Results:** The patient developed rapidly progressive epidermal detachment with severe oral, ocular, and genital mucosal involvement shortly after exposure to trimethoprim/sulfamethoxazole (TMP-SMX). Disease severity was reflected by a SCORTEN score of 5, corresponding to a very high predicted mortality risk. The clinical picture raised concern for both TEN and severe acute GVHD, while histopathological findings favored TEN but were not definitive. Management included systemic corticosteroids, intravenous immunoglobulin, ruxolitinib, and intensive supportive care. The patient gradually re-epithelialized and recovered without long-term sequelae. **Conclusions:** This case underscores the diagnostic difficulty of distinguishing TEN from severe acute GVHD in the early post-transplant period. Careful assessment of drug exposure, clinical evolution, and multidisciplinary evaluation are essential to guide timely and appropriate management.

## 1. Introduction

Allogeneic hematopoietic stem cell transplantation (allo-HSCT) is a potentially curative treatment for various hematologic malignancies but is accompanied by substantial infectious and immunologic risks. Post-transplant patients routinely receive broad antimicrobial prophylaxis, including trimethoprim/sulfamethoxazole (TMP-SMX) for *Pneumocystis jirovecii* pneumonia [1]. However, TMP-SMX is also associated with severe cutaneous adverse reactions (SCARs), including Stevens–Johnson syndrome (SJS) and toxic epidermal necrolysis (TEN) [2,3,4]. Pharmacovigilance and epidemiologic data show that TMP-SMX is associated with a disproportionate signal for SJS/TEN compared with several other commonly used antibiotics, as well as that the risk of serious rash is several times higher than for other antimicrobials such as azithromycin or amoxicillin/clavulanate [5,6].

SCARs are life-threatening T-cell-mediated hypersensitivity reactions in which drug antigens are presented by specific human leukocyte antigen (HLA) molecules, leading to the clonal activation of cytotoxic T cells and massive keratinocyte apoptosis [2,4,7]. In TEN, CD8^+^ cytotoxic T cells in lesional skin and blister fluid recognize the culprit drug in an HLA class I restricted manner and induce epidermal necrosis via perforin/granzyme pathways [4,8,9].

Clinically, TEN presents with rapidly progressive confluent erythema, blistering, extensive epidermal detachment, and mucosal involvement. It is associated with high acute and long-term morbidity and mortality, particularly in immunocompromised patients such as allo-HSCT recipients [2,10,11]. After allo-HSCT, TEN may closely mimic severe acute skin graft-versus-host disease (stage IV), both clinically and histologically, creating a diagnostic dilemma. Careful assessment of drug exposure, organ involvement, and histopathology is essential to distinguish TEN from GVHD and to promptly withdraw the offending agent [2,11,12].

The following case describes an allo-HSCT recipient who received TMP-SMX and subsequently developed a rapidly evolving, exfoliative rash. Based on the clinical course, the distribution and severity of skin detachment, mucosal involvement, and the exclusion of alternative etiologies, TMP-SMX-induced TEN was considered the most probable diagnosis. This case highlights the challenges of recognizing TMP-SMX-associated TEN in the post-transplant setting and emphasizes the need for heightened vigilance and early intervention in this high-risk population.

## 2. Case Presentation

A 64-year-old male with newly diagnosed acute myeloid leukemia (AML) was referred for treatment in June 2024. His medical history was notable for arterial hypertension and dyslipidemia. He had no known drug allergies, no significant family history, and no harmful habits and was not receiving any regular outpatient medications at diagnosis.

Initial laboratory evaluation revealed marked leukocytosis (WBC 215 × 10^9^/L), anemia (hemoglobin 82 g/L), and thrombocytopenia (platelets 22 × 10^9^/L). Bone marrow aspiration demonstrated 86% blasts. Flow cytometry identified a myeloid blast population expressing CD45, cytoplasmic MPO, CD13, CD33, CD34, CD117, and HLA-DR, with an absence of lymphoid markers. Cytogenetic and molecular testing revealed inv(16)(p13;q22) with CBFB–MYH11 fusion, consistent with AML.

Induction chemotherapy with standard “7+3” (cytarabine and anthracycline) was initiated but failed to achieve complete remission, with 15% residual blasts on post-induction marrow assessment. Salvage therapy with venetoclax and azacitidine was started in August 2024. After three cycles, bone marrow examination demonstrated 4% blasts, consistent with morphologic remission.

Given the disease course, the patient proceeded to allo-HSCT. A 10/10 HLA-matched unrelated donor was identified. Reduced intensity conditioning consisted of fludarabine and busulfan, combined with anti-thymocyte globulin (ATG). GVHD prophylaxis included methotrexate and cyclosporine, with a target trough concentration of 200–250 ng/mL. On 26 November 2024, peripheral blood stem cells were infused, delivering 9.2 × 10^6^ CD34^+^ cells/kg. The immediate transplant procedure was uncomplicated.

Platelet engraftment, defined as a platelet count > 20 × 10^9^/L without transfusion support, was achieved on day +21, and neutrophil engraftment (absolute neutrophil count > 0.5 × 10^9^/L) was achieved on day +24. Donor chimerism analysis performed on day +27 demonstrated mixed chimerism, with 63% donor CD34^+^ cells and 90% donor CD3^+^ cells. At that time, bone marrow evaluation revealed no detectable genetic abnormalities and cytological examination showed <5% blasts.

**Figure 1:**
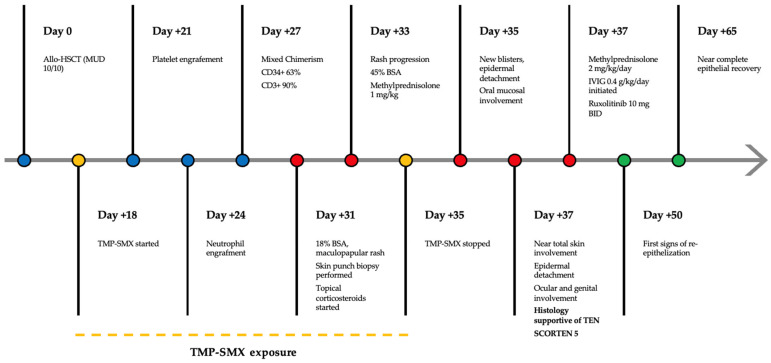
A timeline of major clinical events following allo-HSCT, including TMP-SMX exposure, the onset and progression of cutaneous manifestations, diagnostic evaluation, therapeutic interventions, and recovery. Colors indicate transplant-related events (blue), TMP-SMX exposure and treatment milestones (yellow), disease progression (red), and recovery (green).

The early post-transplant course was marked by mucositis, gastrointestinal symptoms, and febrile neutropenia beginning on day +10. Broad-spectrum antibiotic therapy was initiated; however, blood and site cultures remained negative. Due to persistent fever in the setting of absolute pancytopenia, antimicrobial coverage was progressively escalated to include tigecycline, TMP-SMX, acyclovir, and liposomal amphotericin B, with TMP-SMX initiated on day +18 after allo-HSCT. Following the escalation of antimicrobial therapy, the patient became afebrile and inflammatory markers decreased, with C-reactive protein declining from a peak value of 240 mg/L. A single recurrent febrile episode occurred on day +30, at which time CRP remained low at 8 mg/L. The key clinical events, including TMP-SMX exposure, cutaneous progression, treatment escalation, and recovery, are summarized in Figure 1.

**Figure 2:**
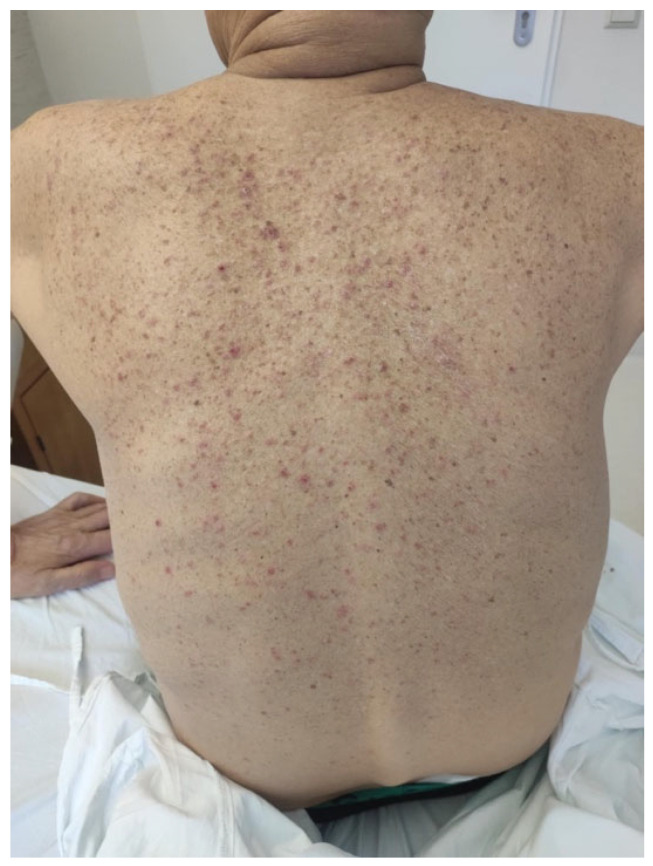
An early cutaneous rash on day +31 post-transplant, showing confluent erythematous papules and plaques involving approximately 18% BSA, predominantly on the back.

**Figure 3:**
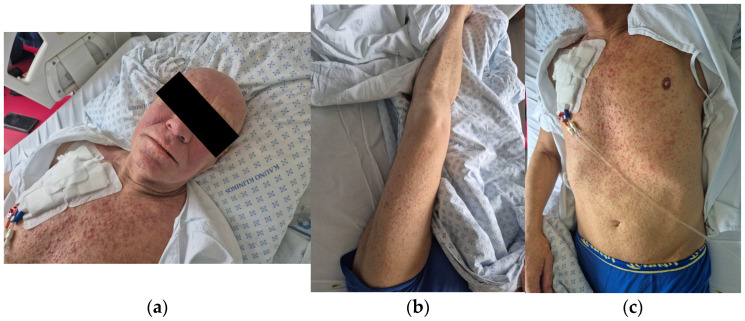
Rapid rash progression by day +33 post-transplant, involving approximately 45% BSA. (**a**) Anterior trunk and upper chest. (**b**) Lower-extremity involvement. (**c**) Confluent anterior trunk and abdominal rash without blistering or epidermal detachment.

On day +31 post-transplant, the patient developed a diffuse erythematous maculopapular rash, composed of numerous discrete papules with areas of confluence, predominantly involving the trunk and covering approximately 18% of the body surface area 13 days after TMP-SMX initiation (Figure 2). At that time, the absolute neutrophil count was 3.5 × 10^9^/L and the platelet count was 42 × 10^9^/L. No mucosal involvement was observed. Acute cutaneous graft-versus-host disease was suspected. A punch biopsy of the skin was performed, and topical corticosteroid therapy was initiated. Despite treatment, the rash progressed rapidly in extent, and by day +33 skin, involvement had expanded to approximately 45% of the body surface area, with the rash having spread to the trunk and lower extremities, while dorsal involvement remained unchanged (Figure 3). At this stage, no epidermal detachment or blistering was observed and the clinical presentation was still considered consistent with severe acute cutaneous graft-versus-host disease, prompting the initiation of systemic corticosteroid therapy with intravenous methylprednisolone at a dose of 1 mg/kg/day.

**Figure 4:**
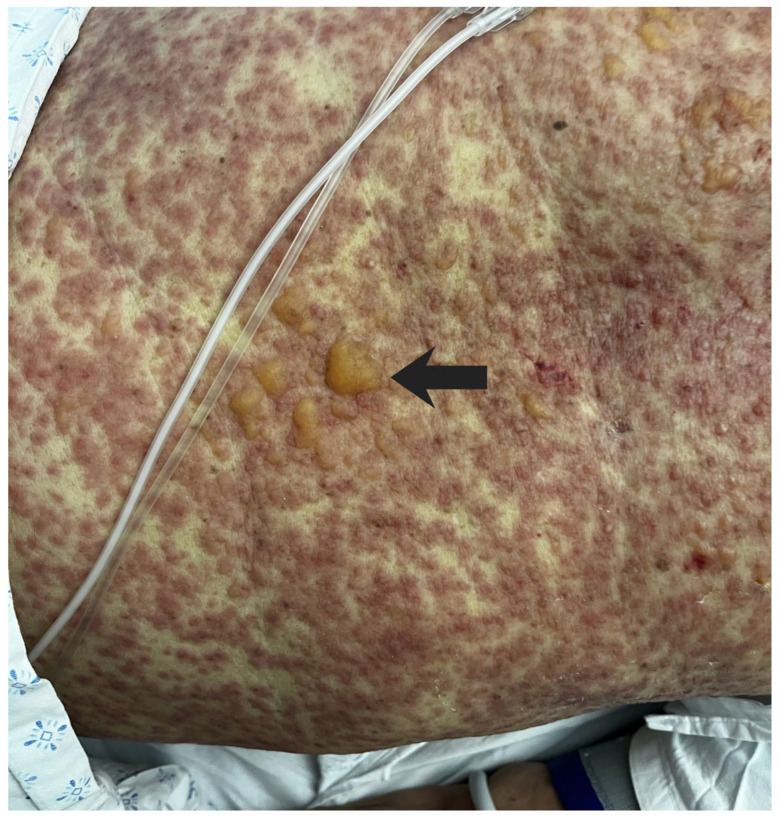
New blister formation on day +35 post-transplant. The arrow indicates a tense subepidermal blister on diffusely erythematous abdominal skin.

On day +35 post-transplant, the clinical appearance of the skin rash changed markedly, with the development of new tense subepidermal blisters on a diffusely erythematous background, most prominently involving the abdominal skin (Figure 4). This represented a clear morphological progression compared with prior examinations. At this time, the patient reported significant oral mucosal pain, while ocular and genital mucosal involvement was not yet present. The patient remained afebrile; however, inflammatory markers began to rise, with the C-reactive protein level increasing to 35 mg/L.

No gastrointestinal manifestations suggestive of acute GVHD were observed. The patient remained free of diarrhea, nausea, vomiting, and abdominal pain, while bilirubin and liver enzyme levels remained within normal limits throughout the period of cutaneous disease progression.

Given the emergence of blistering lesions and the evolving clinical picture, the differential diagnosis was broadened to include Stevens–Johnson syndrome/toxic epidermal necrolysis in addition to severe acute cutaneous graft-versus-host disease. TMP-SMX was discontinued, and high-dose systemic corticosteroid pulse therapy was initiated.

**Figure 5:**
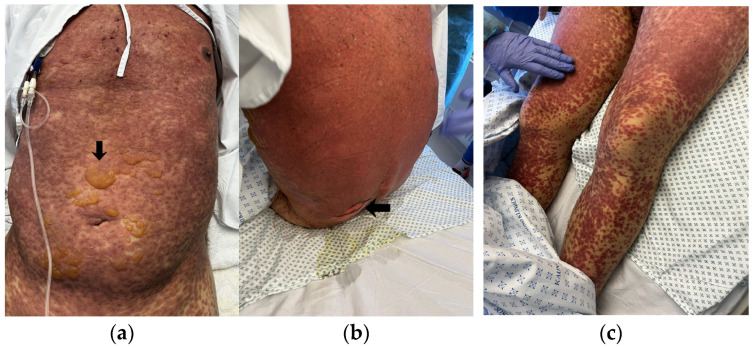
Peak cutaneous involvement on day +37 post-transplant. (**a**) Extensive anterior trunk involvement with tense bullae; the arrow indicates a prominent blister. (**b**) Epidermal detachment and erosions of the lower back/gluteal region; the arrow indicates denuded skin. (**c**) Diffuse erythematous eruption involving both lower extremities.

By day +37 post-transplant, the patient’s condition had progressed to near-total skin involvement, with extensive tense bullae, areas of epidermal detachment, and widespread erosions affecting the trunk and lower extremities (Figure 5). The Nikolsky sign was positive upon examination of the trunk. Mucosal involvement became prominent, with severe oral pain; ocular involvement, characterized by conjunctival hyperemia, conjunctival ulcerations, and bulbar conjunctival erosions; and severe genital pain, with small erosions on the glans penis.

At this time, the patient was subfebrile (maximum temperature 37.7 °C). Laboratory evaluation showed a C-reactive protein level of 31 mg/L, an absolute neutrophil count of 6.4 × 10^9^/L, and a platelet count of 50 × 10^9^/L. Renal function was mildly impaired with a serum creatinine level of 130 µmol/L and a urea level of 14.6 mmol/L.

Following multidisciplinary team evaluation, toxic epidermal necrolysis was favored over acute GVHD with stage 4 skin involvement based on the extent of epidermal detachment, severe multisite mucosal involvement, a positive Nikolsky sign, and clinical evolution. High-dose systemic therapy was initiated, including intravenous immunoglobulin at 0.4 g/kg/day, the escalation of systemic corticosteroids to methylprednisolone at 2 mg/kg/day, and the addition of ruxolitinib at a dose of 10 mg twice daily, as severe acute GVHD could not be completely excluded at that time.

Supportive care was managed jointly by the transplant, dermatology, ophthalmology, urology, and wound-care teams. The patient received intravenous crystalloids with potassium and magnesium replacement guided by daily fluid balance, renal function, and electrolyte monitoring. Blood glucose was monitored regularly and corrected with insulin as needed. Because oral intake was limited by mucosal involvement and overall disease severity, parenteral nutrition was initiated. Analgesia was adjusted according to pain intensity and included paracetamol and morphine. The patient was managed in protective isolation, with surveillance cultures from the skin, oral mucosa, and ocular surface repeated every 48 h. Broad-spectrum antimicrobial therapy was adjusted according to microbiological findings and susceptibility testing, particularly after *Pseudomonas aeruginosa* was isolated from non-blood sites. Cutaneous care was performed once to twice daily under sterile conditions using gentle irrigation with sterile water, local antiseptic treatment with octenidine when needed, paraffin net dressings with topical silver sulfadiazine, sterile secondary dressings, and external warming measures. Crusted lesions on the face, lips, nose, ears, and eyelids were treated with petrolatum, while loose exfoliated epidermis was gently removed when necessary. Ophthalmological care included eyelid cleansing with normal saline four times daily, artificial tears six times daily, dexamethasone and levofloxacin eye drops four times daily, and tobramycin ointment twice daily. Because of urethral involvement, a Foley catheter was inserted after urologic evaluation.

Based on the extent and severity of cutaneous involvement on day +37, disease severity was assessed using the SCORTEN prognostic score, which was calculated prospectively. The patient had a SCORTEN of 5, indicating a very high predicted mortality risk, with the following criteria present: age > 40 years, underlying malignancy, heart rate > 120 beats per minute, epidermal detachment involving >10% of the body surface area, and hyperglycemia with serum glucose > 250 mg/dL (14 mmol/L).

**Figure 6:**
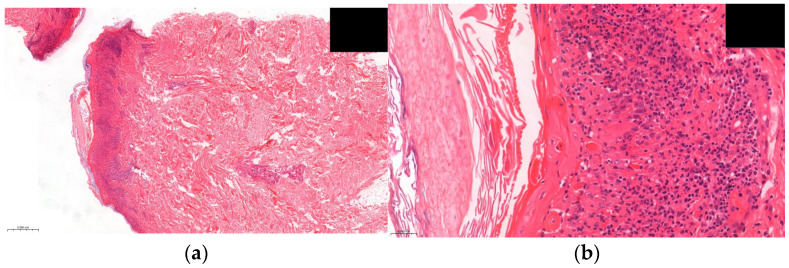
Skin biopsy findings consistent with epidermal necrolysis. (**a**) Low-power H&E view showing epidermal necrosis and subepidermal blister formation. (**b**) Higher-power view showing necrotic keratinocytes and inflammatory infiltrates.

The histopathological examination of a 5 mm punch biopsy obtained from the back demonstrated extensive epidermal necrosis with numerous necrotic keratinocytes, in some areas involving the full thickness of the epidermis, accompanied by subepidermal blister formation (Figure 6). The dermis showed mild perivascular lymphocytic infiltration with occasional eosinophilic and neutrophilic granulocytes. A mixed leukocytic infiltrate was also present within the epidermis and superficial dermis, predominantly with a perivascular distribution and only limited peri-adnexal involvement. Overall, the histopathological findings were compatible with epidermolytic bullous skin changes characteristic of Stevens–Johnson syndrome/toxic epidermal necrolysis. However, given the post-transplant context, severe acute graft-versus-host disease could not be completely excluded and correlation with the clinical presentation was recommended.

During the period of extensive skin barrier disruption, cultures obtained from the skin, oral cavity, urethra, and ocular surface grew *Pseudomonas aeruginosa*. Blood cultures remained negative. The isolate was susceptible to meropenem and amikacin, and targeted antimicrobial therapy was administered accordingly, with meropenem continued until day +64 and amikacin until day +65 post-transplant.

**Figure 7:**
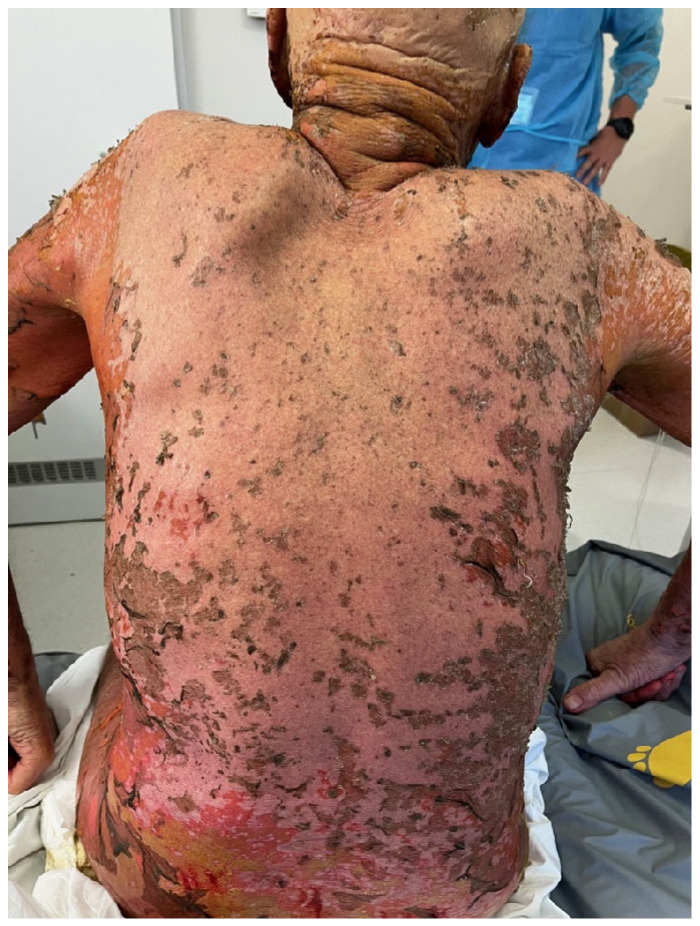
The early re-epithelialization phase observed around day +50, with the partial restoration of epidermal integrity and reduced erosions.

**Figure 8:**
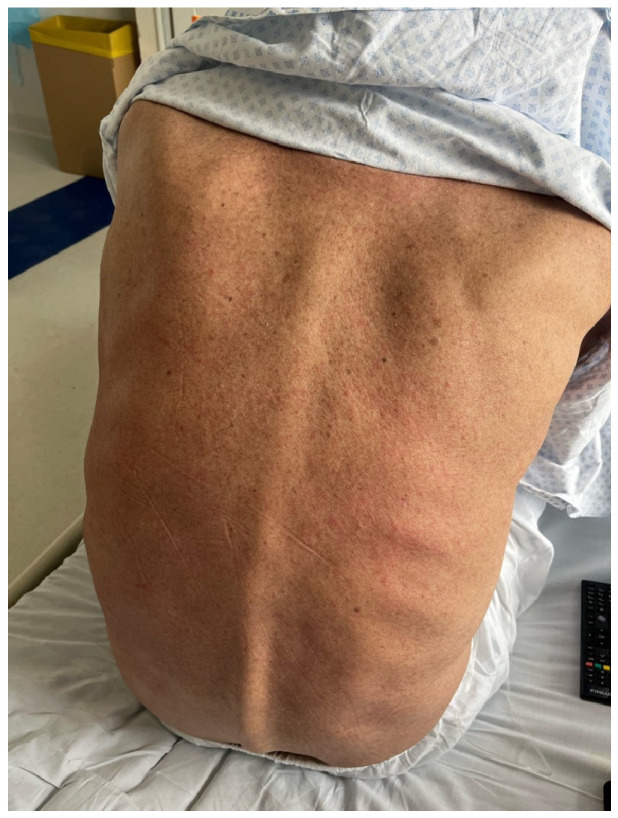
Near-complete epithelial recovery by day +65.

The first signs of re-epithelialization were observed between days +50 and +55 post-transplant (Figure 7). Progressive improvement followed, with near-complete re-epithelialization achieved by day +65 (Figure 8). The patient survived the episode of toxic epidermal necrolysis and recovered without permanent cutaneous, ocular, or genital sequelae.

## 3. Discussion

This case highlights how difficult it can be to distinguish between drug-induced toxic epidermal necrolysis and severe acute graft-versus-host disease in the early post-transplant period, where both can present with very similar features. TEN is a rare, life-threatening severe cutaneous adverse reaction characterized by extensive epidermal necrosis and detachment involving more than 30% of the body surface area [13,14]. It occurs in about 0.4–1.2 cases per million persons per year, but, despite its rarity, mortality remains high, being commonly around 25–35% and even higher in patients with significant comorbidities or immunosuppression [14,15,16]. Most cases are drug-induced, typically triggered by medications such as antibiotics, antiepileptics, NSAIDs, and other high-risk drugs, with drugs implicated in roughly 80–90% of SJS/TEN cases [17].

In the setting of allogeneic hematopoietic stem cell transplantation (allo-HSCT), distinguishing TEN from other post-transplant complications is particularly challenging. Transplant recipients are exposed to numerous drugs, including chemotherapy, antimicrobial prophylaxis, and intensive immunosuppression, all of which can trigger severe cutaneous adverse reactions or mimic them [18]. Profound post-transplant immune dysregulation further complicates the interpretation of new rashes and mucosal disease [18,19]. Crucially, TEN, in this context, may closely resemble severe acute cutaneous graft-versus-host disease, a common and potentially life-threatening complication of allo-HSCT, making accurate clinicopathologic differentiation essential for appropriate management [20,21].

The clinical overlap between TEN and severe acute cutaneous GVHD is substantial, particularly in transplant recipients. Severe forms of acute GVHD can closely mimic TEN [20,22]. Both conditions may begin with diffuse erythematous maculopapular rashes that can rapidly progress to generalized involvement with erythroderma, blistering, and widespread epidermal detachment, often accompanied by mucosal disease and systemic complications [20,22,23,24]. In transplant patients, these syndromes frequently arise within weeks after transplantation or after changes in immunosuppression, creating major diagnostic uncertainty [20,24]. Key clinical and histopathological features that may help to distinguish severe acute cutaneous GVHD from SJS/TEN in allo-HSCT recipients are summarized in Table 1. Selected published reports describing TEN after allo-HSCT are summarized in Table 2. These cases highlight the diagnostic challenges of differentiating TEN from acute GVHD and demonstrate the variable triggers, treatment strategies, and outcomes reported in the literature.

In this case, TMP-SMX was favored as the most likely culprit because the clinical course fit well with drug-induced epidermal necrolysis. The rash appeared 13 days after TMP-SMX was started, progressed while the drug was still being administered, and improved after the withdrawal of the suspected agent together with intensive supportive and immunomodulatory treatment. To make this assessment more objective, we applied the ALDEN algorithm, which is specifically designed for drug causality assessment in SJS/TEN. TMP-SMX received an ALDEN score of 5, corresponding to probable causality. Although severe acute GVHD remained an important competing diagnosis, the rapid progression to extensive epidermal detachment, prominent multisite mucosal involvement, and overall disease evolution were more consistent with TEN.

Acute GVHD remained a serious consideration throughout the early course, particularly because the eruption initially resembled severe cutaneous GVHD. However, the disease pattern became less typical for GVHD as the case evolved. During the period of rapid skin progression, the patient had no diarrhea, nausea, vomiting, abdominal pain, bilirubin elevation, or liver enzyme abnormalities, making gastrointestinal or hepatic GVHD unlikely. Mixed donor chimerism on day +27 (CD34^+^ 63%, CD3^+^ 90%) confirmed early post-transplant immune reconstitution but did not provide a specific explanation for the cutaneous syndrome. The timing shortly after engraftment was compatible not only with acute GVHD but also with drug-induced SJS/TEN. Importantly, the eruption progressed despite corticosteroid escalation and evolved into extensive epidermal detachment with prominent oral, ocular, and genital mucosal involvement, a pattern that favored TEN over isolated severe cutaneous GVHD.

Histopathologically, TEN and severe acute cutaneous GVHD share key features, including vacuolar damage of the basal layer, keratinocyte apoptosis, and confluent epidermal necrosis, sometimes with subepidermal blister formation and variable dermal inflammatory infiltrates, limiting the specificity of routine biopsy [24,25]. TEN-like acute GVHD represents the severe end of cutaneous GVHD and may closely resemble drug-induced TEN, both clinically and microscopically [20,22,26]. In general, TEN is more often characterized by widespread epidermal necrosis with a relatively sparse inflammatory infiltrate, whereas severe cutaneous GVHD may show more prominent interface dermatitis, satellitosis, adnexal involvement, and lymphocytic infiltration [20,22,25,26]. Because of this overlap, clinicopathologic correlation remains essential, including timing after transplant, recent drug exposure, gastrointestinal or hepatic GVHD involvement, and detailed histologic assessment. In our case, histology was more consistent with drug-induced epidermal necrolysis but did not definitively exclude severe acute GVHD.

In the present case, several features supported a diagnosis of TEN rather than severe acute cutaneous GVHD. The clinical course was marked by an abrupt transition from a maculopapular rash to widespread blistering and epidermal detachment within a short time frame. The development of tense bullae, a positive Nikolsky sign, and rapid progression to near-total skin involvement are characteristic features of TEN. Additionally, the presence of severe multisite mucosal involvement, including oral, ocular, and genital lesions, strongly favored TEN, as mucosal involvement in GVHD is typically less extensive and evolves differently. The timing and pattern of progression, together with the absence of other organ manifestations typically associated with severe GVHD, further supported this interpretation.

Exposure to TMP-SMX represented a key etiologic factor in this case. TMP-SMX is one of the most commonly implicated drugs in TEN and is widely used for *Pneumocystis jirovecii* pneumonia prophylaxis in transplant recipients [5,6]. The time between TMP-SMX administration and the onset of symptoms in our patient is consistent with the known latency period of drug-induced TEN, which typically ranges from 1 to 3 weeks after exposure [5,11]. In addition, immunocompromised patients may have an increased susceptibility to severe drug reactions, further supporting the role of TMP-SMX as the likely trigger in this case.

The management of TEN in the post-transplant setting remains complex and is not standardized. In this case, treatment included high-dose systemic corticosteroids, intravenous immunoglobulin (IVIG) and ruxolitinib, along with intensive supportive care. While the use of systemic corticosteroids and IVIG in TEN remains controversial, these therapies are commonly employed in severe cases, particularly when differentiation from GVHD is uncertain [27,28,29]. Ruxolitinib, a JAK 1/2 inhibitor, was introduced because severe acute GVHD could not be completely excluded at the time of clinical deterioration and because of its established role in steroid-refractory GVHD. Although its immunomodulatory effects, mediated through the inhibition of JAK-STAT-dependent cytokine signaling, may theoretically have contributed to controlling the severe inflammatory response, its specific role in TEN remains uncertain [30,31]. Given the concurrent withdrawal of TMP-SMX, escalation of corticosteroids, administration of IVIG, and intensive supportive care, the individual contribution of ruxolitinib to clinical recovery cannot be determined. Supportive care, including meticulous wound management, infection control, fluid and electrolyte balance, and multidisciplinary involvement, remains the cornerstone of TEN management and is critical for patient survival.

The severity of disease was reflected by a SCORTEN of 5. In the original SCORTEN validation study, a score of ≥5 was associated with a predicted mortality of >90% (95% confidence interval, 55.5–99.8%) [32]. However, SCORTEN has not been specifically validated in allo-HSCT recipients and should therefore be interpreted here as a marker of severe disease rather than a precise individualized mortality estimate. Despite this, the patient achieved complete recovery without long-term sequelae. This favorable outcome likely reflects early recognition of the evolving clinical picture, prompt discontinuation of the suspected offending agent, and initiation of aggressive systemic and supportive therapy. The absence of bloodstream infection, despite documented colonization with *Pseudomonas aeruginosa* and the implementation of appropriate targeted antimicrobial therapy, may also have contributed to the positive outcome.

This case highlights the significant diagnostic challenges associated with severe cutaneous reactions in the early post-transplant period. Distinguishing between TEN and severe acute cutaneous GVHD is critical, as the underlying pathophysiology, management strategies, and prognostic implications differ substantially. Clinicians should maintain a high index of suspicion for drug-induced TEN in patients receiving high-risk medications such as TMP-SMX, particularly when rapid clinical deterioration, blister formation, and extensive mucosal involvement are observed. Early multidisciplinary evaluation, including dermatologic and histopathologic assessment, is essential to guide appropriate management.

**Table 1:** The clinical and histopathological features useful in distinguishing severe acute cutaneous GVHD from SJS/TEN in allo-HSCT recipients [2,4,18,26,33].

Feature	Severe Acute Cutaneous GVHD	SJS/TEN
Mechanism	Donor T-cell activation against the recipient cells	Hypersensitivity reaction with cytotoxic T lymphocytes and natural killer cell activation
Typical trigger	Allo-HSCT with donor alloreactivity	Medications most common, infections less common
Timing	Usually within first 100 days after allo-HSCT	Usually 1–3 weeks after culprit drug exposure
Initial rash	Maculopapular eruption, often involving the face, palms, and soles	Painful erythematous or dusky lesions that may rapidly become confluent
Mucosal involvement	In TEN-like acute GVHD, bullae, epidermal detachment, and mucosal involvement may closely mimic SJS/TEN	Common and often prominent: oral, ocular, and genital
Nikolsky sign	May be present in severe cases	Often positive
Systemic manifestations	May be accompanied by gastrointestinal or hepatic GVHD, including diarrhea, abdominal pain, cholestasis, or bilirubin elevation	Fever, malaise, pain, mucosal involvement, fluid loss, infection risk, and multiorgan complications in severe cases
Laboratory findings	No specific diagnostic laboratory marker. Bilirubin or liver enzyme abnormalities may support hepatic GVHD when present	No specific diagnostic laboratory marker; SCORTEN variables may reflect severity, including renal dysfunction, hyperglycemia, bicarbonate decrease, and tachycardia
Histology	Interface dermatitis with basal vacuolization, apoptotic keratinocytes, satellitosis, and more prominent lymphocytic/interface inflammation	Full-thickness epidermal necrosis with subepidermal blistering and usually sparse dermal inflammatory infiltrate
Expected therapeutic response	Usually managed by the escalation of immunosuppression. Response to steroids or GVHD-directed therapy supports GVHD	Requires the withdrawal of the suspected culprit drug. Improvement after drug withdrawal supports SJS/TEN
Key diagnostic clue	Concurrent gastrointestinal or hepatic GVHD may support diagnosis	Recent high-risk drug exposure, rapid detachment, and prominent mucosal involvement

**Table 2:** Selected published reports of TEN after allo-HSCT [12,20,22,34,35,36,37].

Study	Age/Sex	Underlying Malignancy	Transplant Type (Donor/HLA Match)	Concurrent GVHD	Suspected Trigger	Treatment	Outcome
Hilgendorf et al., 2007 [12]	-	Multiple myeloma	MUD (HLA-matched)	No active GVHD reported	TMP-SMX, levofloxacin, amoxicillin/clavulanate	Steroids, IVIG, supportive care	Recovered
Macedo et al., 2014 [20]	23/F	T-cell lymphoma	Mismatched unrelated donor	Grade IV GI GVHD	Cefepime	Steroids, IVIG, supportive care	Died
Gomulka et al., 2014 [34]	67/F	CLL	Allo-HSCT	Grade IV intestinal GVHD	Voriconazole	IVIG, supportive care	Recovered
Chen et al., 2018 [35]	67/M	AML	Allo-HSCT	Uncertain	TMP-SMX, NSAIDs	Etanercept, supportive care	Recovered
Morishige et al., 2019 [36]	73/M	ATLL	Haploidentical HSCT	GVHD/TEN overlap	Mogamulizumab, Lenalidomide	Steroids, IVIG, supportive care	Recovered
Vera et al., 2022 [22] (Case 1)	58/M	AML	Allo-HSCT	Acute GVHD present	TMP-SMX ± voriconazole	IVIG, etanercept, supportive care	Died
Vera et al., 2022 [22] (Case 2)	67/F	MDS	MUD HSCT	Acute GVHD present	TMP-SMX	Infliximab, Cyclophosphamide, supportive care	Died
Chromik et al., 2024 [37]	24/M	Refractory Hodgkin lymphoma	MUD 10/10	Initially diagnosed as grade I aGVHD	Prior nivolumab exposure	Burn center care, amniotic membranes	Recovered
Present case	64/M	AML	MUD 10/10	Acute GVHD considered but not confirmed	TMP-SMX	Steroids, IVIG, supportive care, ruxolitinib	Recovered

## 4. Conclusions

Toxic epidermal necrolysis should be considered in allo-HSCT recipients presenting with rapidly progressive cutaneous rashes, particularly in the context of high-risk drug exposure such as TMP-SMX. Differentiating TEN from severe acute cutaneous GVHD remains challenging due to significant clinical and histopathological overlap, but features such as abrupt blistering, extensive mucosal involvement, and disease evolution may aid distinction. Early recognition and prompt withdrawal of the offending agent, together with aggressive supportive and immunomodulatory therapy, are critical for improving outcomes. This case underscores the importance of maintaining diagnostic vigilance in the post-transplant setting, where timely decisions can be lifesaving.

## Data Availability

The original contributions presented in this study are included in the article. Further inquiries can be directed to the corresponding author.

## Abbreviations

The following abbreviations are used in this manuscript:

TEN	Toxic epidermal necrolysis
SJS	Stevens–Johnson syndrome
Allo-HSCT	Allogeneic hematopoietic stem cell transplantation
GVHD	Graft-versus-host disease
TMP-SMX	Trimethoprim/sulfamethoxazole
SCAR	Severe cutaneous adverse reaction
HLA	Human leukocyte antigen
AML	Acute myeloid leukemia
MDS	Myelodysplastic syndrome
CLL	Chronic lymphocytic leukemia
ATLL	Adult T-cell leukemia/lymphoma
MUD	Matched unrelated donor
ATG	Anti-thymocyte globulin
CRP	C-reactive protein
BSA	Body surface area
NSAID	Nonsteroidal anti-inflammatory drug
IVIG	Intravenous immunoglobulin
JAK	Janus kinase
JAK-STAT	Janus kinase–signal transducer and activator of transcription pathway
